# Effects of steroid hormones on lipid metabolism in sexual dimorphism: A Mendelian randomization study

**DOI:** 10.3389/fendo.2022.1119154

**Published:** 2023-01-16

**Authors:** Junzhi Liang, Bowen Zhang, Yannan Hu, Zhijing Na, Da Li

**Affiliations:** ^1^ Center of Reproductive Medicine, Shengjing Hospital of China Medical University, Shenyang, China; ^2^ Key Laboratory of Reproductive and Genetic Medicine (China Medical University), National Health Commission, Shenyang, China; ^3^ Key Laboratory of Reproductive Dysfunction Diseases and Fertility Remodeling of Liaoning Province, Shengjing Hospital of China Medical University, Shenyang, China

**Keywords:** sexual dimorphism, steroid hormones, lipid metabolism, Mendelian randomization study, single-nucleotide polymorphisms

## Abstract

**Background:**

Although the role of steroid hormones in lipid levels has been partly discussed in the context of separate sexes, the causal relationship between steroid hormones and lipid metabolism according to sex has not been elucidated because of the limitations of observational studies. We assessed the relationship between steroid hormones and lipid metabolism in separate sexes using a two-sample Mendelian randomization (MR) study.

**Methods:**

Instrumental variables for dehydroepiandrosterone sulfate (DHEAS), progesterone, estradiol, and androstenedione were selected. MR analysis was performed using inverse-variance weighted, MR-Egger, weighted median, and MR pleiotropy residual sum and outlier tests. Cochran’s Q test, the MR-Egger intercept test, and leave-one-out analysis were used for sensitivity analyses.

**Results:**

The results showed that the three steroid hormones affected lipid metabolism and exhibited sex differences. In males, DHEAS was negatively correlated with total cholesterol (TC), low-density lipoprotein cholesterol (LDL-C), and apolipoprotein B (*P* = 0.007; *P* = 0.006; *P* = 0.041, respectively), and progesterone was negatively correlated with TC and LDL-C (*P* = 0.019; *P* = 0.038, respectively). In females, DHEAS was negatively correlated with TC (*P* = 0.026) and androstenedione was negatively correlated with triglycerides and apolipoprotein A (*P* = 0.022; *P* = 0.009, respectively). No statistically significant association was observed between the estradiol levels and lipid metabolism in male or female participants.

**Conclusions:**

Our findings identified sex-specific causal networks between steroid hormones and lipid metabolism. Steroid hormones, including DHEAS, progesterone, and androstenedione, exhibited beneficial effects on lipid metabolism in both sexes; however, the specific lipid profiles affected by steroid hormones differed between the sexes.

## 1 Introduction

Lipid metabolism differs between males and females; these sex differences are known as sexual dimorphism. Compared with males, females have been reported to have higher concentrations of total cholesterol (TC), low-density lipoprotein cholesterol (LDL-C), and high-density lipoprotein cholesterol (HDL-C) and lower concentrations of triglycerides and apolipoprotein B (ApoB) (1). These differences may be due to multiple factors, such as sex hormones (2–4) and sex chromosomes (5, 6).

Steroid hormones are derived from cholesterol synthesis and regulate lipid metabolism. Several studies have reported that steroid hormones might have different effects on lipid metabolism in according to sex (2, 3). Published randomized controlled trials have suggested that oral supplementation with dehydroepiandrosterone (DHEA) hormone results in lower serum LDL-C levels in men, but no significant effect in women (7, 8). In addition, sex hormones significantly affect the function and deposition of adipose tissues (9, 10). Premenopausal women accumulate more subcutaneous fat, whereas men tend to have more visceral fat (11).

While the role of steroid hormones in lipid levels has been partly discussed in the context of separate sexes, the causal relationship between steroid hormones and lipid metabolism according to sex has not been elucidated because of the limitations of observational studies that cannot definitively identify causality. Mendelian randomization (MR) is a method that uses genetic variations associated with exposures to evaluate potential causality with outcomes (12). MR analysis is protected from confounding and reverse causality due to the random assignment of alleles at the time of conception, as well as the natural direction from genetic variation to phenotype (13, 14).

In this study, we conducted sex-stratified MR analysis to reveal the causal relationship between steroid hormones and lipid metabolism according to sex. We screened 17 single-nucleotide polymorphisms (SNPs) associated with 4 steroid hormones from the Leipzig Research Center for Civilization Diseases. The relationship of the 17 SNPs with lipid metabolism outcomes in the UK Biobank (UKBB, largest cohort in Europe) study participants were further evaluated.

## 2 Methods

### 2.1 Study design

**Figure 1:**
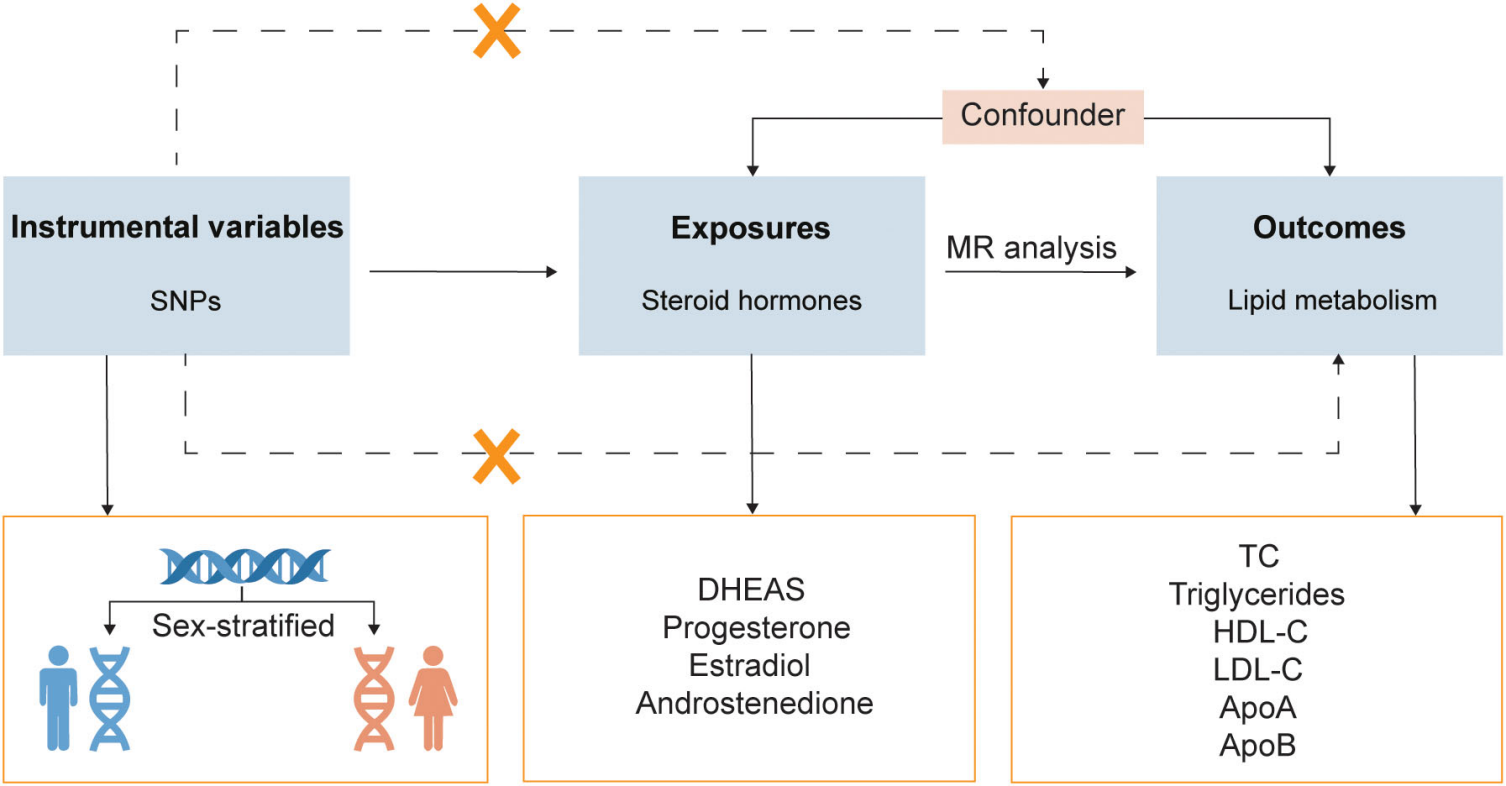
Study design overview. SNPs, single-nucleotide polymorphisms; MR, Mendelian randomization; DHEAS, dehydroepiandrosterone sulfate; HDL-C, high-density lipoprotein cholesterol; LDL-C, low-density lipoprotein cholesterol; TC, total cholesterol; ApoA, apolipoprotein A; ApoB, apolipoprotein B.

A two-sample MR design was utilized to evaluate the causal relationship between steroid hormones and lipid metabolism in both sexes (**Figure 1**). The summary statistics used were publicly available; therefore, no ethical approval was required.

### 2.2 Data of exposure

The exposure data were subjected to sex-stratified analysis (including Modus ALL, Modus MALE, and Modus FEMALE). Steroid hormones were derived from two cohorts from the Leipzig Research Center for Civilization Diseases: LIFE-Adult and LIFE-Heart. These two cohorts consisted of 17,000 participants, all of whom were from Leipzig, Germany. Genetic associations were adjusted for age and body mass index (BMI) in all modus and adjusted for sex in Modus ALL in the genome-wide association study (GWAS). We extracted 17 SNPs that were independently and strongly correlated with hormones in one or more modus as instrumental variables (*P* < 5 × 10^-8^, r2 > 0.5, mean *F*-statistics > 10) (15). Each SNP was examined using the Phenoscanner database to eliminate confounding factors. To ensure MR quality, we removed SNPs that were highly correlated with the outcomes. Finally, 17 SNPs were screened as instrumental variables for 4 hormones: dehydroepiandrosterone sulfate (DHEAS), progesterone, estradiol, and androstenedione.

### 2.3 Data of outcome

Sex-stratified molecular data on lipid metabolism were obtained from the UKBB database (16). In a large, population-based, prospective cohort study, the UKBB recruited more than 500,000 participants aged 40-69 years between 2006 and 2010 to measure and record both phenotypic and genotypic data. The six lipid metabolism molecules included TC, triglycerides, HDL-C, LDL-C, apolipoprotein A (ApoA), and ApoB.

### 2.4 Two-sample MR analysis

MR analysis uses genetic variation as an instrumental variable, and the following criteria were applied in this study: (i) strongly associated with steroid hormones, (ii) independent of confounding factors, and (iii) correlation with lipid metabolism, only through steroid hormones (17, 18). When only one SNP was present, we utilized the Wald ratio method, and when multiple SNPs were present, inverse-variance weighted (IVW) was utilized as the prime method to evaluate causality. Because IVW provides MR estimates by combining each Wald ratio of multiple SNPs, it shows the greatest statistical power among all the MR methods (19). In addition, we utilized the weighted median and MR-Egger methods for the sensitivity analyses.

### 2.5 Sensitivity analysis

In this study, we used Cochran’s Q test to quantify heterogeneity among the instrument variables (20). The MR-Egger intercept test (21) and leave-one-out analysis were used to describe the potential horizontal pleiotropy and assess the robustness of the results. Furthermore, MR pleiotropy residual sum and outlier (MR-PRESSO) was used to examine potential outliers and rectify pleiotropy by removing outliers, if required (22).

The “TwoSampleMR” and “MRPRESSO” R packages in RStudio version 4.2.1 were used for our study. *P* values were calculated bilaterally, and *P* < 0.05 was considered statistically significant.

## 3 Results

### 3.1 Steroid hormones and lipid metabolism in total population

**Figure 2:**
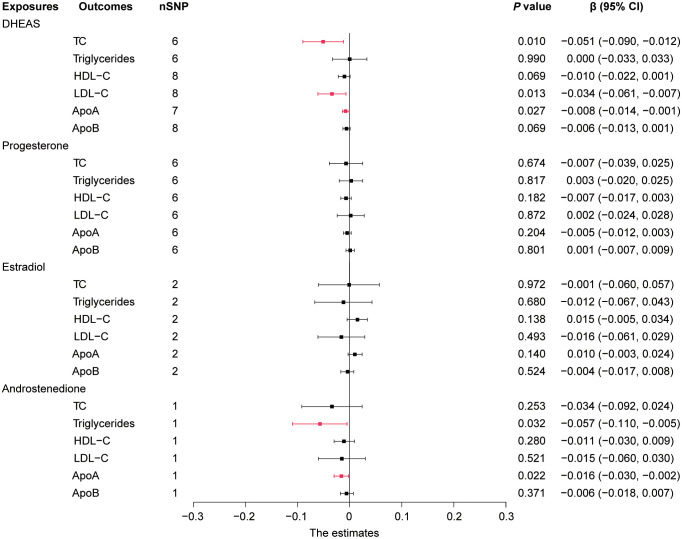
Forest plot of IVW estimates in total population. DHEAS, dehydroepiandrosterone sulfate; nSNP, number of single-nucleotide polymorphisms; HDL-C, high-density lipoprotein cholesterol; LDL-C, low-density lipoprotein cholesterol; 95% CI, 95% confidence interval; IVW, inverse-variance weighted; TC, total cholesterol; ApoA, apolipoprotein **A**; ApoB, apolipoprotein **B**.

In this study, we performed MR analysis with the 17 screened SNPs (**Supplementary Tables 1**-**5**) and utilized IVW as the prime method to evaluate causality. DHEAS was negatively correlated with TC (β = -0.051, 95% CI: -0.090 to -0.012, *P* = 0.010) and LDL-C (β = -0.034, 95% CI: -0.061 to -0.007, *P* = 0.013) in the total population. After the deletion of outliers, DHEAS also showed a negative correlation with ApoA levels (*P* = 0.027). Additionally, a negative correlation was observed between androstenedione and triglycerides (*P* = 0.032) and ApoA (*P* = 0.022) levels in the total population (**Figure 2**). All the sensitivity analyses supported these results (**Supplementary Table 6**).

In short, in the total population, an increase in DHEAS correlated with a decrease in TC, LDL-C, and ApoA, and an increase in androstenedione correlated with a decrease in triglycerides and ApoA. In contrast, estradiol levels were not significantly correlated with lipid levels in the total population.

Notably, MR analysis revealed the presence of significant sex differences in the effects of some steroid hormones on serum lipids, as described below.

### 3.2 Steroid hormones and lipid metabolism in males

**Figure 3:**
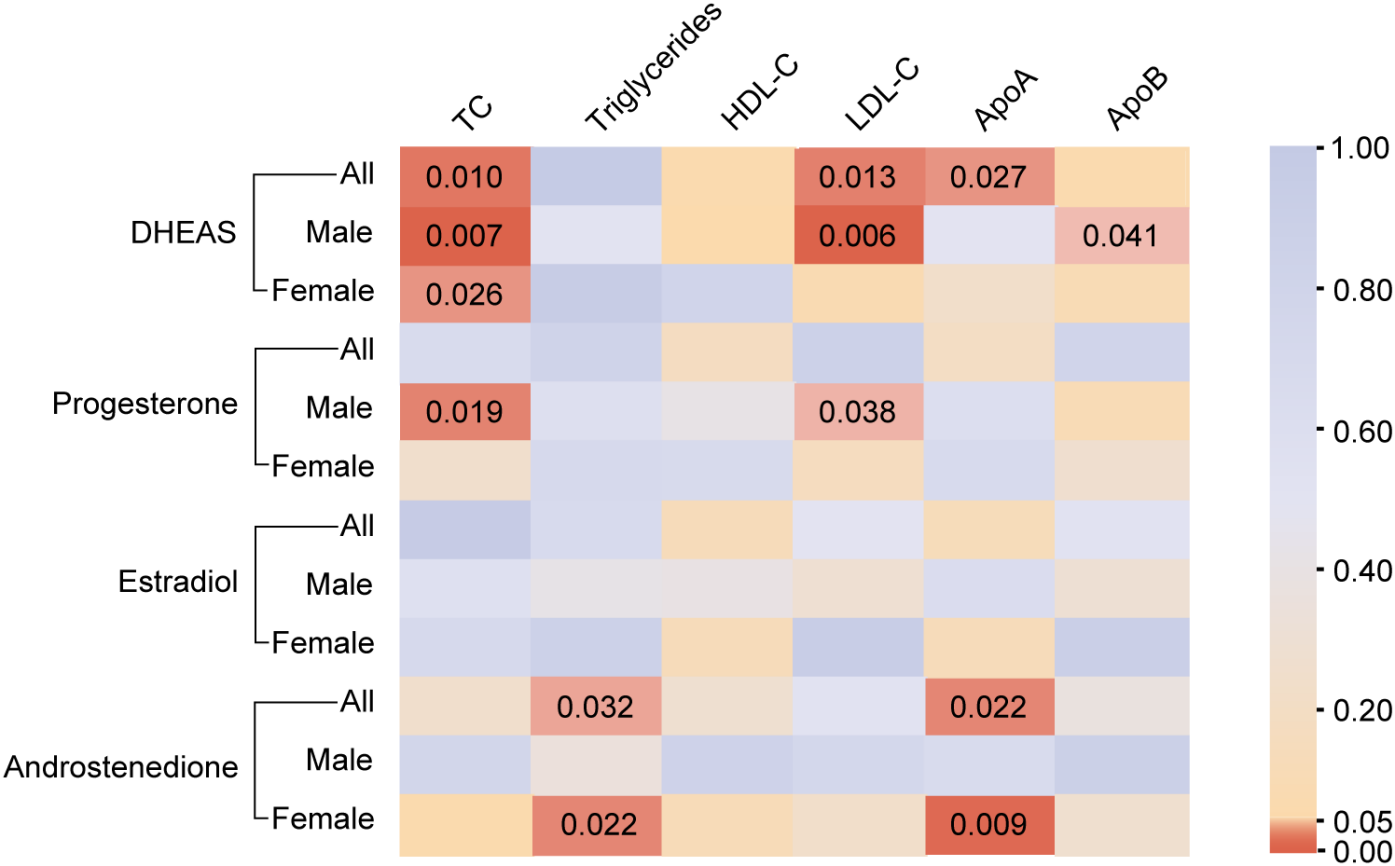
The causal association of steroid hormones on lipid metabolism. The color of each block represents the *P* values of the IVW or Wald ratio method. The brown indicates *P* < 0.05, and the yellow/blue indicates *P* > 0.05. P < 0.05 was considered to indicate a significant correlation. All, total population includes males and females; DHEAS, dehydroepiandrosterone sulfate; HDL-C, high-density lipoprotein cholesterol; LDL-C, low-density lipoprotein cholesterol; IVW, inverse-variance weighted; TC, total cholesterol; ApoA, apolipoprotein A; ApoB, apolipoprotein B.

**Figure 4:**
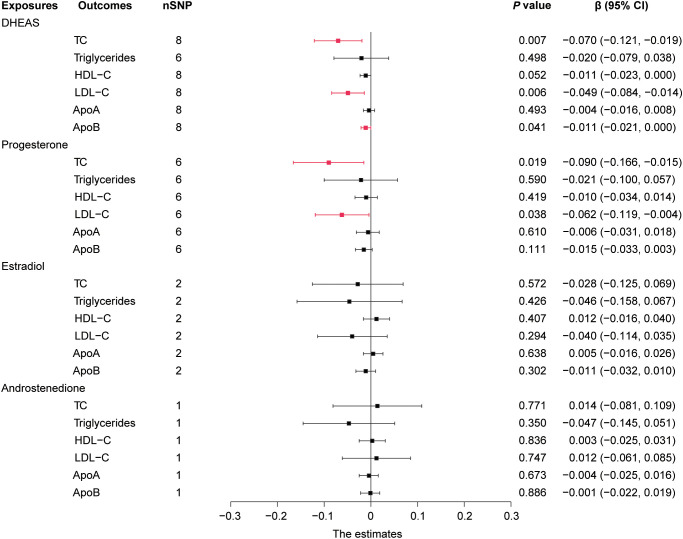
Forest plot of IVW estimates in males. DHEAS, dehydroepiandrosterone sulfate; nSNP, number of single-nucleotide polymorphisms; HDL-C, high-density lipoprotein cholesterol; LDL-C, low-density lipoprotein cholesterol; 95% CI, 95% confidence interval; IVW, inverse-variance weighted; TC, total cholesterol; ApoA, apolipoprotein A; ApoB, apolipoprotein B.

In males, genetically predicted elevated DHEAS was associated with a trend toward decreased TC (β = -0.070, 95% CI: -0.121 to -0.019, *P* = 0.007), LDL-C (β = -0.049, 95% CI: -0.084 to -0.014, *P* = 0.006), and ApoB (β = -0.011, 95% CI: -0.021 to 0.000, *P* = 0.041). MR analysis also revealed that elevated progesterone levels were associated with a trend toward decreased TC (*P* = 0.019) and LDL-C (*P* = 0.038), as shown in **Figures 3**, **4** and **Supplementary Table 7**. Meanwhile, we conducted MR analysis with the weighted median and MR-Egger, and the β values of all three methods were negative, exhibiting the same trend (**Table 1**).

**Table 1:** MR estimates for the association between steroid hormones and lipid metabolism in males.

Outcomes	DHEAS				Progesterone			
	nSNP	β	95% CI	*P* value	nSNP	β	95% CI	*P* value
TC								
IVW	8	-0.070	-0.121 to -0.019	0.007	6	-0.090	-0.166 to -0.015	0.019
Weight median	8	-0.058	-0.115 to 0.000	0.050	6	-0.093	-0.195 to 0.009	0.075
MR-Egger	8	-0.023	-0.119 to 0.073	0.651	6	-0.194	-0.395 to 0.007	0.132
LDL-C								
IVW	8	-0.049	-0.084 to -0.014	0.006	6	-0.062	-0.119 to -0.004	0.038
Weight median	8	-0.030	-0.073 to 0.012	0.160	6	-0.069	-0.141 to 0.002	0.057
MR-Egger	8	-0.011	-0.074 to 0.052	0.743	6	-0.113	-0.267 to 0.041	0.222
ApoB								
IVW	8	-0.011	-0.021 to 0.000	0.041	6	-0.015	-0.033 to 0.003	0.111
Weight median	8	-0.007	-0.018 to 0.004	0.222	6	-0.018	-0.037 to 0.002	0.073
MR-Egger	8	-0.003	-0.022 to 0.017	0.800	6	-0.029	-0.080 to 0.022	0.326

DHEAS, dehydroepiandrosterone sulfate; TC, total cholesterol; LDL-C, low-density lipoprotein cholesterol; ApoB, apolipoprotein B; nSNP, number of single-nucleotide polymorphisms; IVW, inverse-variance weighted; 95% CI, 95% confidence interval; MR, Mendelian randomization.

**Figure 5:**
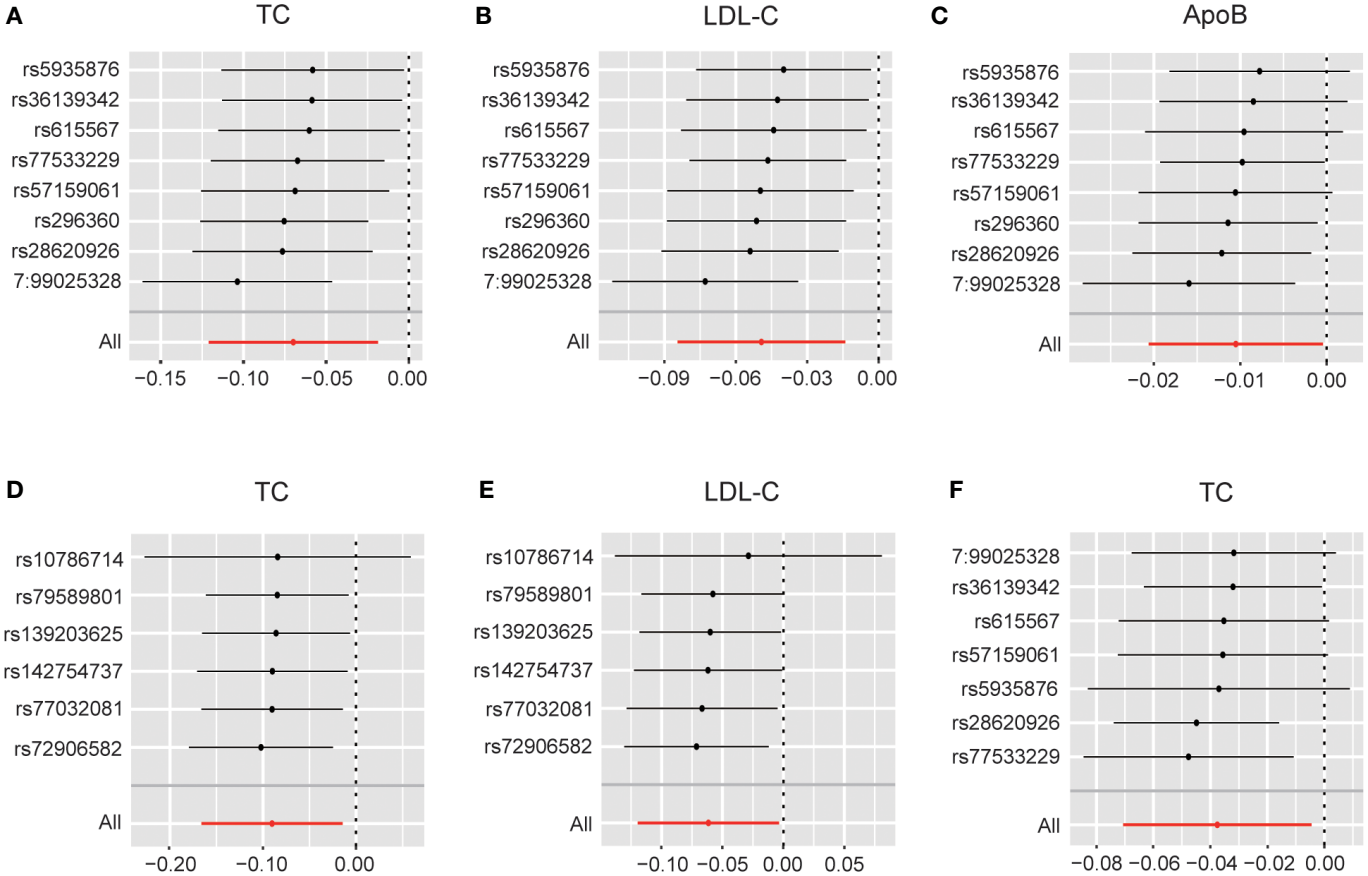
**(A)** Leave-one-out analysis plots for DHEAS on total cholesterol in males; **(B)** Leave-one-out analysis plots for DHEAS on LDL-C in males; **(C)** Leave-one-out analysis plots for DHEAS on apolipoprotein B in males; **(D)** Leave-one-out analysis plots for progesterone on total cholesterol in males; **(E)** Leave-one-out analysis plots for progesterone on LDL-C in males; **(F)** Leave-one-out analysis plots for DHEAS levels on total cholesterol in females. LDL-C, low-density lipoprotein cholesterol; TC, total cholesterol; ApoB, apolipoprotein B.

These results are supported by a range of sensitivity analyses (**Supplementary Table 8**). Cochran’s Q test and the MR-Egger intercept test did not detect apparent heterogeneity or pleiotropy, and MR-PRESSO did not detect outliers. Furthermore, the leave-one-out test showed no potentially influential SNPs (**Figures 5A–E**). These results confirm the statistical significance of this correlation.

Overall, in males, an increase in DHEAS correlated with a decrease in TC, LDL-C, and ApoB, and an increase in progesterone correlated with a decrease in TC and LDL-C. In contrast, androstenedione and estradiol were not significantly correlated with lipids in males.

### 3.3 Steroid hormones and lipid metabolism in females

In females, the initial genetically predicted DHEAS and TC levels showed no statistically significant correlation. However, repeated MR analysis with an outlier removed by MR-PRESSO showed that elevated DHEAS was associated with a trend toward decreased TC levels (β = -0.038, 95% CI: -0.071 to -0.005, *P* = 0.026). We also performed MR analysis separately using the weighted median (*P* = 0.034) and MR-Egger’s method (*P* = 0.507). The β values of the three methods were negative, showing the same trend. The leave-one-out test showed no potentially influential SNPs (**Figure 5F**). All the sensitivity analyses supported the above results (**Supplementary Table 9**), confirming the statistical significance of this correlation.

**Figure 6:**
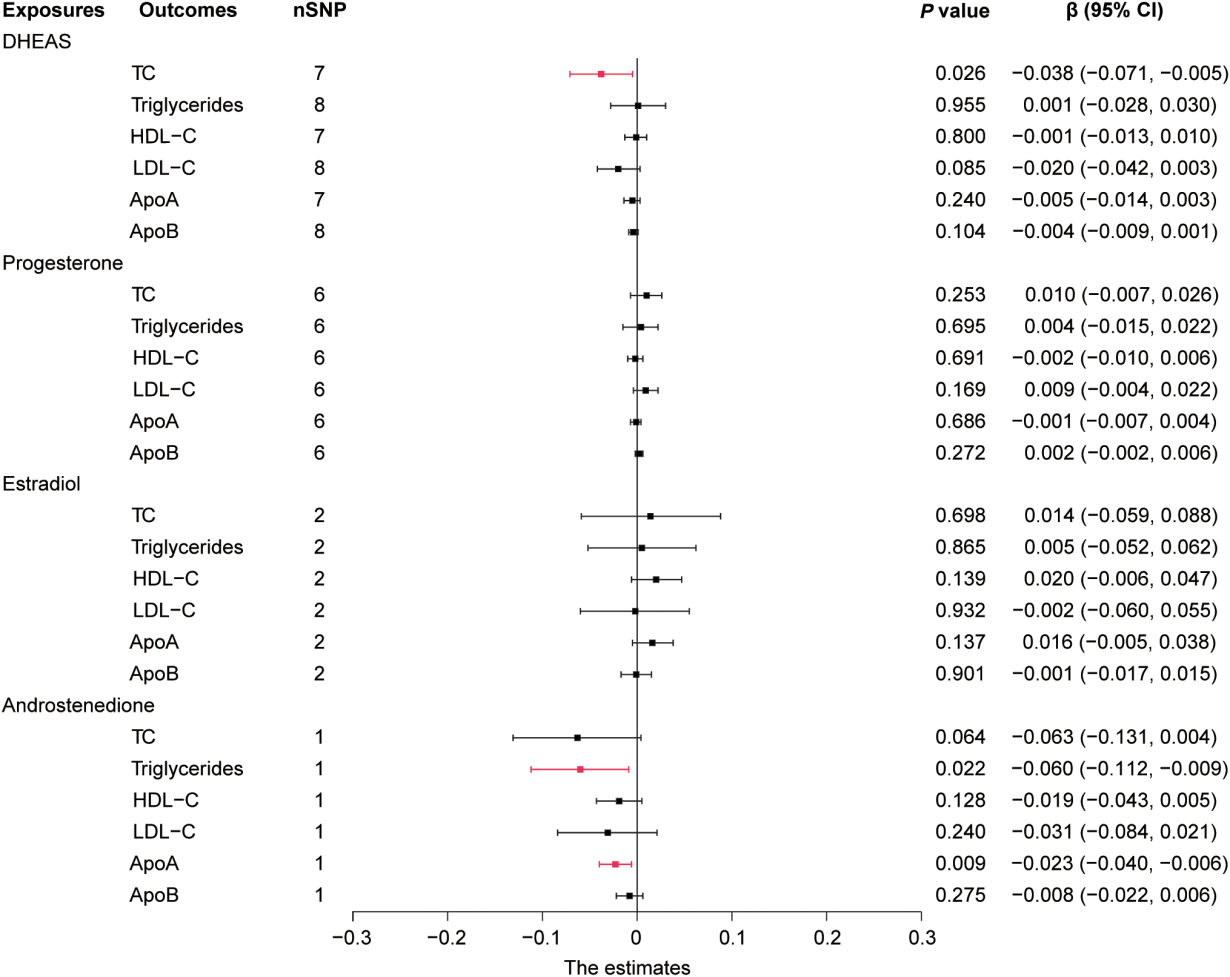
Forest plot of IVW estimates in females. DHEAS, dehydroepiandrosterone sulfate; nSNP, number of single-nucleotide polymorphisms; HDL-C, high-density lipoprotein cholesterol; LDL-C, low-density lipoprotein cholesterol; 95% CI, 95% confidence interval; IVW, inverse-variance weighted; TC, total cholesterol; ApoA, apolipoprotein A; ApoB, apolipoprotein B.

In addition, MR analysis revealed that elevated androstenedione was associated with a trend toward decreased triglycerides (*P* = 0.022) and ApoA (*P* = 0.009) (**Figure 6**). The results of the MR analysis by Wald ratio showed that this association was statistically significant. However, due to the limited number of SNPs, further MR and sensitivity analyses were not performed, and the results require further clinical epidemiological support.

Overall, in females, an increase in DHEAS was correlated with a decrease in TC and an increase in androstenedione was correlated with a decrease in triglycerides and ApoA. In contrast, neither of the two hormones, progesterone or estradiol, displayed a statistically significant association with lipids in females.

## 4 Discussion

To our knowledge, this is the first study to use MR analysis to systematically investigate sex differences in the causal relationship between steroid hormones and lipid metabolism. Our study showed that the three steroid hormones exhibit sex differences in their effects on lipid metabolism. Some of the relationships between steroid hormones and lipids identified in this study are supported by epidemiological and mechanistic studies, while others are novel and require confirmation by further epidemiological studies and future mechanistic exploration.

DHEAS showed a statistically significant correlation with lipid levels in both males and females, and this association exhibited differences between the sexes. In males, elevated DHEAS levels were associated with lower concentrations of TC, LDL-C, and ApoB. In females, DHEAS showed a weaker negative correlation with TC. The cause of sexual dimorphism in lipid changes due to DHEAS is unclear. However, it has been shown that DHEA supplementation leads to sex differences in changes of several sex hormones including serum total testosterone (23), which may further affect sex hormone binding globulin (SHBG) level. SHBG plays a key role in the association of sex hormones with lipids and influences lipid regulation (24, 25), which may account for this difference. Previous studies have shown that androgen DHEA or DHEAS supplementation was negatively associated with serum lipids overall, but this relationship differed significantly by sex in randomized controlled trials, as shown in **Table 2**. These differences may be due to the older or diseased participants, small number of participants, short duration of treatment, different dosing concentrations, different methods of measuring lipids, ethnic differences, and conversion of DHEA to other sex hormones. These possible confounding factors and reverse causality highlight the importance of MR study. In addition, the conclusions drawn from the MR analysis were in accordance with the findings of several epidemiological studies. Lower levels of DHEAS were associated with a higher incidence of cardiovascular disease and cardiovascular disease-related mortality in males and females (35–38) which might result from low DHEAS-associated hyperlipidemia. However, the exact mechanism underlying this regulatory effect remains unclear and requires further research.

**Table 2:** Randomized controlled trials of DHEA or DHEAS supplementation in males and females.

TC		LDL-C		HDL-C		ApoB		Triglycerides		References
Male	Female	Male	Female	Male	Female	Male	Female	Male	Female	
NS	NS	NS	NS	NS	NS	?	?	↓	NS	(26)
NS	NS	?	?	?	?	NS	NS	NS	NS	(27)
?	?	NS	NS	NS	↓	?	?	NS	NS	(28)
↓	?	?	?	↓	?	?	?	NS	?	(29)
NS	?	↓	?	NS	?	NS	?	NS	?	(7)
NS	?	NS	?	NS	?	?	?	↓	?	(30)
?	?	?	↓	?	↑	?	?	?	↓	(31)
?	↓	?	↓	?	↓	?	?	?	↓	(32)
?	↓	?	NS	?	↓	?	?	?	NS	(8)
?	↓	?	NS	?	NS	?	?	?	NS	(33)
?	NS	?	NS	?	NS	?	?	?	NS	(34)

The symbol, “?” indicates that the correlation is unknown; the symbol, “↑” indicates an increase in lipids after DHEA or DHEAS supplementation; the symbol, “↓” indicates a decrease in lipids after DHEA or DHEAS supplementation.

DHEA, dehydroepiandrosterone; DHEAS, dehydroepiandrosterone sulfate; TC, total cholesterol; LDL-C, low-density lipoprotein cholesterol; HDL-C, high-density lipoprotein cholesterol; ApoB, apolipoprotein B; NS, not statistically significant (P > 0.05).

Another surprising finding was that estradiol was not significantly correlated with lipids in both males and females, and this result should be interpreted with caution. Previous studies have shown that people who were supplemented with estrogen by oral or transdermal means, including postmenopausal females (39), post-hysterectomy females (40, 41), males with prostate cancer (42), and male-to-female transsexuals (43), presented with altered lipids. Notably, different means of estrogen supplementation can lead to different changes in serum lipid levels (39, 44). Oral estrogen has been reported to result in lower LDL-C and higher HDL-C levels, whereas transdermal estrogen leads to lower TC levels (39). This difference may be associated with the “first-pass effect” in the liver. Mechanistic studies also support the aforementioned modulatory effects of estrogen on lipids. Estrogen has been reported to achieve control of lipid metabolism by mediating three receptors: estrogen receptor alpha, estrogen receptor beta, and G protein-coupled estrogen receptor (45). Nevertheless, estrogen supplementation only partially mimics estrogenic action, and this supraphysiological amount of hormone supplementation may not necessarily help us understand the mechanism of lipid regulation by these hormones in their physiological state.

Alterations in estrogen in physiological states did not significantly affect lipid homeostasis. Most studies have reported that women have minimal changes in serum lipid concentrations throughout the menstrual cycle, with only a small decrease in LDL-C concentrations during the luteal phase (46). Dyslipidemia associated with menopause is largely due to aging, rather than the loss of ovarian function (47, 48). In addition, studies have shown that women have a lower risk of cardiovascular disease and a later age at first onset than men (49). Historically, this difference has been ascribed to the protective effects of endogenous estrogen. Nevertheless, modern research refutes this simplistic explanation and argues that this association cannot be inferred to causality (50). Furthermore, studies have shown that the use of conjugated equine estrogen does not affect the incidence of coronary heart disease in postmenopausal women (51). These seemingly conflicting conclusions demonstrate the complexity of the relationship between estradiol and lipids. Reviewing the available studies and in conjunction with our findings, we cautiously concluded that the correlation between estradiol and lipid metabolism was not statistically significant.

In addition, our MR analysis provides novel insights into the relationship between progesterone, androstenedione, and lipids. We found that progesterone was negatively correlated with serum TC and LDL-C levels in males but had no correlation with lipid metabolism in females. In contrast, androstenedione was negatively correlated with triglycerides and ApoA in females, but not with lipid metabolism in males. Previous studies on the relationships between progesterone, androstenedione, and lipids in males and females are lacking, and this MR analysis provides a novel idea, but more mechanistic and epidemiological studies are needed to validate it.

Compared to previous studies, our study has the advantages of sex-stratification and increased variety of the analyzed steroid hormones. This MR analysis represented steroid hormones by screening instrumental variables, reducing errors due to BMI and age, measuring steroid hormones and lipids, and other factors, and yielding more reliable conclusions. This study had several limitations. First, all GWASs data were obtained from European populations; therefore, our findings should be cautiously extended to other populations. Second, since the effects of steroid hormones on lipid metabolism in different sexes are complex, contradictions between some observational studies or the lack of corresponding studies lead to some conclusions from the MR analysis lacking sufficient support from observational studies. Third, these SNPs were selected by linkage disequilibrium variants that had an r2 of greater than 0.5, which may reduce the credibility of the results. Fourth, the causal relationships between steroid hormones including estradiol, androstenedione and lipids were not performed for sensitivity analyses due to the limitation of the number of SNPs, which may have an impact on the results.

## 5 Conclusions

In this study, we identified sex-specific causal networks of steroid hormone levels and lipid metabolism using MR analysis. Genetically predicted steroid hormones, including DHEAS, progesterone, and androstenedione, exhibited beneficial effects on lipid metabolism in both sexes; however, the specific lipid profiles affected by steroid hormones differed between the sexes. Sexual dimorphism is a critical but often overlooked factor. Understanding the differences in the influence of steroid hormones on lipid metabolism across sexes may provide strategies for designing sex-specific treatments, which is important for reducing the side effects of steroid hormones therapy and for precise therapeutic dosing.

## Data availability statement

Publicly available datasets were analyzed in this study. This data can be found here: UK Biobank (https://www.ukbiobank.ac.uk/).

## Author contributions

JL, DL, and ZN contributed to the manuscript writing and data analysis. BZ participated in the data collection and analysis. JL and YH conducted the image production and manuscript editing. DL and ZN designed the study and reviewed and supervised the manuscript. All authors contributed to the article and approved the submitted version.
